# Astrocyte-mediated short-term synaptic depression in the rat hippocampal CA1 area: two modes of decreasing release probability

**DOI:** 10.1186/1471-2202-12-87

**Published:** 2011-08-24

**Authors:** My S Andersson, Eric Hanse

**Affiliations:** 1 Institute of Neuroscience and Physiology, Gothenburg University, Göteborg, Sweden Box 432, Medicinaregatan 11, 405 30 Göteborg, Sweden

## Abstract

**Background:**

Synaptic burst activation feeds back as a short-term depression of release probability at hippocampal CA3-CA1 synapses. This short-term synaptic plasticity requires functional astrocytes and it affects both the recently active (< 1 s) synapses (post-burst depression) as well as inactive neighboring synapses (transient heterosynaptic depression). The aim of this study was to investigate and compare the components contributing to the depression of release probability in these two different scenarios.

**Results:**

When tested using paired-pulses, following a period of inactivity, the transient heterosynaptic depression was expressed as a reduction in the response to only the first pulse, whereas the response to the second pulse was unaffected. This selective depression of only the first response in a high-frequency burst was shared by the homosynaptic post-burst depression, but it was partially counteracted by augmentation at these recently active synapses. In addition, the expression of the homosynaptic post-burst depression included an astrocyte-mediated reduction of the pool of release-ready primed vesicles.

**Conclusions:**

Our results suggest that activated astrocytes depress the release probability via two different mechanisms; by depression of vesicular release probability only at inactive synapses and by imposing a delay in the recovery of the primed pool of vesicles following depletion. These mechanisms restrict the expression of the astrocyte-mediated depression to temporal windows that are typical for synaptic burst activity.

## Background

The probability of release (P_r_) is a fundamental property of synapses that is regulated by presynaptic activity (short-term synaptic plasticity) [1] and by modulatory transmitters acting on presynaptic receptors [2-4]. P_r _at rest (after seconds of inactivity) varies substantially among synapses [5] and is determined by two independent factors. One is the number of vesicles primed for release and thus potentially available for release by a single action potential, the primed pool. The other is the probability of releasing one primed vesicle (P_ves_) [6,7]. Repeated activation at short intervals, resulting in residual elevated calcium in the presynaptic terminal between activations, will change P_ves_, rapidly deplete the primed pool, and prime new vesicles in a calcium-dependent manner [8]. During high-frequency activation P_r _is rather determined by the rate at which new vesicles can become available for release [7,9]. Thus, factors determining P_r _differ depending on whether the presynaptic terminal has been recently active, or not, and modulatory transmitters may modulate P_r _differently when synapses are active compared to following a period of rest [10].

We have found that activation of astrocytes by a short synaptic burst negatively modulates release probability at CA3-CA1 glutamate synapses [11]. From a period of hundreds of milliseconds to seconds after a short synaptic burst, P_r _is reduced in the recently active synapses (postburst depression, PBD). This PBD is absent when strongly buffering calcium in the astrocyte gap junction-coupled network, when inhibiting astrocyte metabolism and early in development when the astrocyte network still not has gained its mature function. This short-term astrocyte-mediated depression is also observed as a reduction of P_r _in inactive neighboring synapses (transient heterosynaptic depression, tHeSD) [12]. Although P_r _is depressed in the PBD and in tHeSD it is unclear if these depressions are based on the same mechanism. One obvious difference between the PBD and the tHeSD is the recent presynaptic activity. In the present study we have therefore compared the PBD and the tHeSD with respect to estimated changes in P_ves _and primed pool.

## Results

### PBD and tHeSD are associated with different changes in the paired-pulse ratio

**Figure 1:**
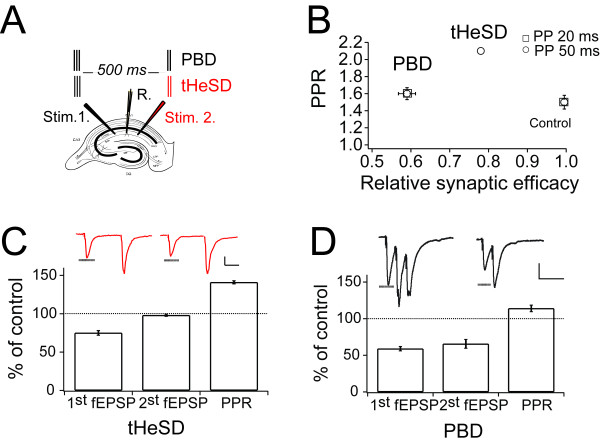
**Homosynaptic postburst depression and transient heterosynaptic depression result in different changes in paired-pulse ratio**. *A*, Schematic representation of the experimental protocol for postburst depression (PBD) and transient heterosynaptic depression (tHeSD). Our standard protocol consisted of a 3-impulse, 50 Hz, burst before a paired-pulse test stimulus applied either homosynaptically (PBD, black), or heterosynaptically (tHeSD, red). *B*, Relationship between relative synaptic efficacy and paired-pulse ratio (PPR) measured with field recordings. Synaptic efficacy is normalized to control, which is set to 1. PPR was evaluated using an interstimulus interval of 20 ms (circles) and 50 ms (squares). Data are represented as mean ± SEM with the following number of experiments; control 50 ms (n = 28), control 20 ms (n = 28), tHeSD 50 ms (n = 29) Error bars are within the symbol. PBD 50 ms (n = 28) and PBD 20 ms (n = 28). *C*, Relative change of the 1^st ^and 2^nd ^fEPSP, as well as PPR in association with the tHeSD. Interstimulus interval 50 ms. On top are example paired-pulse recordings without (left) and with (right) preceding heterosynaptic conditioning, calibration bar 20 ms, 100 μV. *D*, Relative change of the 1^st ^and 2^nd ^fEPSP, as well as PPR in association with the PBD. Interstimulus interval 20 ms. The top inset show example traces from the conditioning three-impulse burst and the test paired-pulse stimulation, calibration bar 20 ms, 100 μV.

A relatively modest conditioning, a 3-impulse (50 Hz) synaptic burst, in the hippocampal CA1 area, results in a substantial short-term homosynaptic (PBD) and heterosynaptic (tHeSD) transient astrocyte-mediated depression, respectively, half a second after the conditioning burst [11,12]. The experimental protocol for the PBD and the tHeSD is schematically shown in Figure 1A. Our standard protocol consisted of a 3-impulse, 50 Hz, burst, 500 milliseconds before a paired-pulse test stimulus applied either homosynaptically (PBD), or heterosynaptically (tHeSD) every 10 seconds (Figure 1A). The control for the heterosynaptic depression was the paired-pulse test preceded 5 s before with a 3-impulse (50 Hz) synaptic burst, every protocol was repeated 18 times.

These two astrocyte-mediated depressions were both associated with increased paired-pulse ratio (PPR), but to different extent. We will use a change in PPR in association with the astrocyte-mediated depressions as an indication of a change in Pr [1] (see also Methods) since we previously have shown that that the PBD is also associated an equal depression reported by the AMPA and by the NMDA EPSCs, as well as by a matching decrease in the 1/CV^2 ^[11]. Here, we first wanted to, in more detail, examine the relationship between the depression and the increased PPR for the PBD and tHeSD, respectively. Under our experimental conditions (e.g. 4 mM calcium and 4 mM magnesium in the extracellular solution), the PPR was about 1.5, both when 20 ms or 50 ms was used as interstimulus interval (Figure 1B). The PPR increased to about 2 in association with the tHeSD, but only to about 1.6 in association with the PBD (2.1 ± 0.03, n = 29 respectively 1.6 ± 0.08 n = 29, p < 0.01), despite the fact that the PBD was expressed as a substantially larger depression of the field EPSP (fEPSP) than the tHeSD (41.5 ± 2%, n = 35 respectively 20 ± 2%, n = 29, p < 0.001) (Figure 1B).

The large increase of the PPR associated with the tHeSD indicates that the second fEPSP in the test paired-pulse protocol should be little affected. Indeed, there was no change of the second fEPSP in association with the tHeSD (Figure 1C). Using a 3-impulse 50 Hz test stimulus (instead of the paired-pulse test) we found that also the third fEPSP of this 3- impulse test stimulus was not depressed (101 ± 6%, n = 7, p > 0. 1). This result indicates that the depression of P_r _in association with the tHeSD is selective for stimuli that occur following a period of rest. In marked contrast to the tHeSD, the second fEPSP in the test paired-pulse protocol was significantly depressed in association with the PBD (34 ± 6%, n = 13, p < 0.01) (Figure 1D). The differential relationship between the amount of depression and the change of the PPR for the PBD and tHeSD, respectively, suggests that different mechanisms are involved in reducing P_r_, raising the question how the PBD and tHeSD may interact.

### Occlusion between PBD and tHeSD

**Figure 2:**
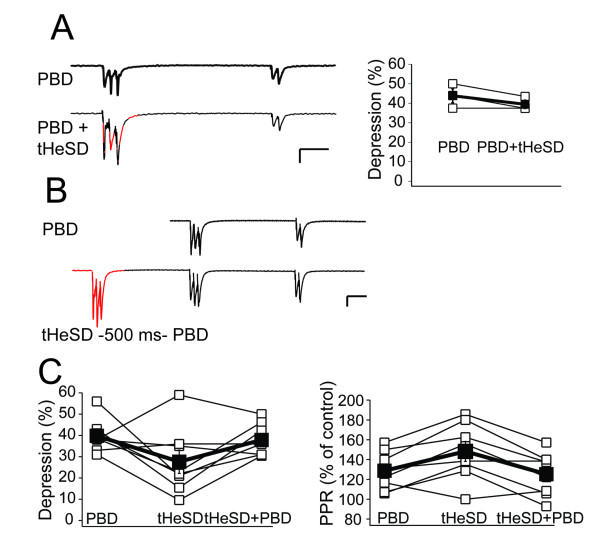
**Interaction between homosynaptic postburst depression and transient heterosynaptic depression**. *A*, Simultaneous homo- and heterosynaptic conditioning (3-impulses, 50 Hz) does not produce more depression than homosynaptic conditioning alone. Left panel shows fESPS recordings of homosynaptic conditioning alone (upper, black trace) and of simultaneous homo- and hetersosynaptic conditioning (lower, red and black trace), calibration bar 100 ms, 200 μV. Right panel summarizes the change in depression by adding heterosynaptic conditioning for three experiments (open squares). The mean change is indicated by the closed square. *B*, Effect of sequential hetero- and homosynaptic conditioning. Left upper panel shows fEPSP recordings of homosynaptic conditioning alone (upper, black trace) and of sequential heterosynaptic - homosynaptic conditioning (lower, red and black trace, 500 ms between conditioning stimuli; hetero- and homosynaptic conditioning indicated in red and black, respectively), calibration bar 100 ms, 100 μV. *C*, The left panel illustrates, for eight experiments, the depression induced by homosynaptic conditioning alone, heterosynaptic conditioning alone and sequential hetero- and homosynaptic conditioning. The corresponding changes in paired-pulse ratio are shown in the right panel.

To test for interaction between the PBD and the tHeSD we applied the 3-impulse burst simultaneously to the two synaptic inputs (Figure 2A) and compared the depression of the fEPSP 500 ms later to the PBD in the same experiment. As demonstrated in Figure 2A the addition of the 3-impulse burst to the other synaptic input did not increase the amount of depression compared to the control PBD, indicating that the homosynaptic conditioning fully elicits the type of depression elicited by the heterosynaptic conditioning (104.4 ± 2%, n = 3, p < 0.1). Alternatively, recent synaptic activity renders synapses resistant to the type of depression elicited by the heterosynaptic conditioning. We next tested to elicit hetero- and homosynaptic depression sequentially by first applying the heterosynaptic conditioning and then, 500 ms later, the homosynaptic conditioning (Figure 2B). The heterosynaptic conditioning elicited a depression of 28 ± 6% (n = 8, p < 0.001) of the fEPSP, associated with an increased PPR of 48 ± 10% (n = 8, p < 0.01) (Figure 2B) (cf. Figure 1). The additional homosynaptic conditioning increased the depression to 38 ± 3%, (n = 8, p < 0.05) but reduced the increase of the PPR to 25 ± 8% (n = 8, p < 0.01) (Figure 2C). These interaction experiments suggest that the homosynaptic PBD consist of two components reducing P_r_, one in common with inactive synapses and one specific for recently active synapses. The depression component specific for the homosynaptic conditioning is associated with decreased PPR, possibly related to facilitation/augmentation induced concomitantly with depletion of primed vesicles by the homosynaptic conditioning.

To elucidate how the homosynaptic and heterosynaptic depressions interact we compared a multiplicative, and an additive relationship with the actual data. In these experiments the average actual PBD was 40 ± 3% (n = 8). An additive relationship between the tHeSD and the remaining depression in the subsequent PBD predicts a total PBD of 48.5 ± 4 (n = 8). A multiplicative relationship predicts a total PBD of 43.8 ± 3.1 (n = 8). Thus, these calculations suggest that a multiplicative interaction and an additive interaction both represent the data reasonable well, but they do not distinguish between the two models.

### PBD is associated with a decreased pool of primed vesicles

**Figure 3:**
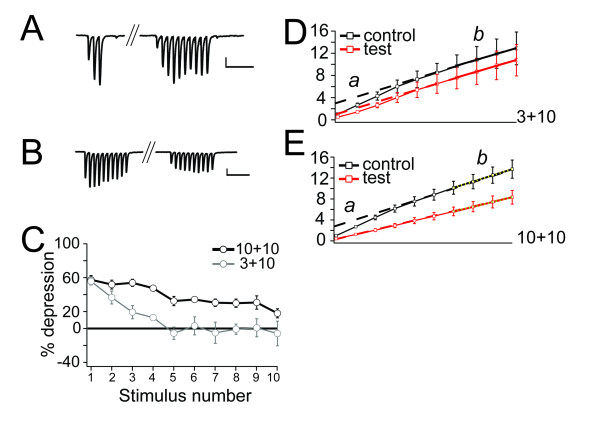
**Homosynaptic postburst depression is associated with a decreased pool of primed vesicles**. *A*, One experiment illustrating the effect of a 3-impulse, 50 Hz, conditioning on a 10-impulse, 50 Hz, test stimuli ("3+10"), calibration bar 100 ms, 50 pA. *B*, One experiment illustrating the effect of a 10-impulse, 50 Hz, conditioning on a 10-impulse, 50 Hz, test stimuli ("10+10"), calibration bar 100 ms, 50 pA. *C*, The amount of depression as a function of stimulus number in the 10-impulse, 50 Hz, test train following 3-impulse (grey line) and 10-impulse (black line) conditioning. *D*, Cumulative EPSC magnitude as a function of stimulus number in a 10-impulse, 50 Hz, train. The cumulative responses during a control train (black) is compared to the cumulative responses during a train evoked 500 ms after a 3-impulse, 50 Hz, conditioning burst (red). EPSCs were normalized with respect to the magnitude of the 1^st ^EPSC in the control train. A linear fit was applied to the last four data points in the cumulative train response and that line was extrapolated to the first stimulus (dashed lines). *E*, Cumulative EPSC magnitude as a function of stimulus number in 10-impulse, 50 Hz, train. The responses during a control train (black) is compared to the responses during a train evoked 500 ms after a 10-impulse, 50 Hz, conditioning train (red). EPSCs were normalized with respect to the magnitude of the 1^st ^EPSC in the control train. A linear fit was applied as above (dashed lines).

In contrast to the tHeSD, which was associated with a selective depression of the first EPSP, also the second EPSP in the paired-pulse protocol was reduced in the PBD (albeit to a lesser extent than the first EPSP). To further examine the consequences of homosynaptic conditioning we used a train of 10 action potentials (at 50 Hz) as a test stimulus, and used whole-cell voltage-clamp recordings to monitor the responses (Figure 3A). Under these conditions the 3-impulse conditioning burst caused a depression of the first EPSC in the train by 56 ± 4% (n = 7, p < 0.001), which is somewhat larger than the depression observed using field recordings and a 2-impulse test stimulus (Figure 1). As shown in Figure 3C, not only the first and second EPSC were depressed, but also the third and fourth, whereas the last six EPSCs in the test train were largely unaffected. Increasing the number of stimuli in the conditioning burst to 10 extended the depression of the test train to all synaptic responses (Figure 3B). A direct comparison of the depressions caused by 3- and 10-impulse conditioning shows that, whereas the 10-impulse conditioning causes an overall larger depression, the depression of the first EPSC (54 ± 4%, n = 7, p < 0.001) is about the same in these two situations (Figure 3C) [11].

The overall response during train stimulation can be used to estimate the relative number of primed vesicles available for release at the onset of the train stimulation [7,13,14]. The rationale behind this estimation is that these vesicles are rapidly consumed by the first few stimuli in the train and priming of new vesicles starts rapidly in the presence of elevated intraterminal calcium. When pre-primed vesicles are consumed and the activity-dependent priming is fully developed, P_r _per stimulus becomes rather constant because the release is matched by the rate of new priming. By plotting the cumulative EPSC amplitude (expressed in units of the 1^st ^EPSC in the control train) as a function of stimulus number (Figure 3D, E), the relative priming rate can be estimated as the slope of the linear late part of the cumulative EPSC - stimulus number relationship. This analysis indicated that the activity-dependent priming rate was unaffected by the 3-impulse conditioning (1.1 ± 0.04 vs 1.1 ± 0.03, n = 7) (Figure 3D), whereas it was reduced by the 10-impulse conditioning (1.2 ± 0.01 vs 0.9 ± 0.003, n = 7, p < 0.01) (Figure 3E).

The number of (primed) vesicles available for release at the onset of the train stimulation can be estimated by subtracting the contribution from activity-dependent priming (recruitment) of new vesicles from the total response during the train. The relative magnitude of the estimate of the pre-primed pool will then depend on when during the train stimulation recruitment is assumed to start. Assuming full recruitment rate already at the first stimulus in the train, an estimate of the pre-primed pool of vesicles can be obtained by the value of the extrapolated regression line at the first stimulus. With this representation of the pre-primed pool, we observed a reduction by 61 ± 0.5% (*n *=, 7, p < 0.001) following the 3-impulse conditioning and by 92 ± 4% (*n *= 7, p < 0.001) following the 10-impulse conditioning. These values are most certainly an overestimate of the depression since the method of back extrapolating will underestimate the pre-primed pool size [cf. 13]. Nevertheless, this analysis indicates that the PBD is associated with a reduction of the pre-primed pool of vesicles and that this reduction increases when increasing the number of stimuli in the conditioning train.

A possible explanation for the similar magnitude of the PBD following a conditioning burst of 3 and 10 impulses (Figure 3C) is that the smaller pool (larger depletion) following the 10 impulse conditioning is compensated for by an increased P_ves_, i.e. facilitation/augmentation [15], thereby maintaining P_r _[16]. A burst consisting of 3-4 impulses, and of 10 impulses at 50 Hz have previously been shown to elicit an augmentation at these CA3-CA1 synapses (single exponential decay with a time constant of about eight seconds) of about 108% and 124% of control, respectively, two seconds after the burst [17]. If an increased facilitation/augmentation counteracts an increased depletion, one would expect that the PBD following the 10-impulse conditioning should be associated with a smaller PPR than the PBD following the 3-impulse conditioning, since facilitation/augmentation is associated with pronounced decrease in PPR [18]. Consistent with a larger depletion (associated with no change in PPR) and a larger facilitation/augmentation (associated with decreased PPR) the PPR was indeed smaller after the 10-impulse (0.9 ± 0.10, n = 7), than after the 3-impulse (1.15 ± 0.10, n = 7), conditioning (p < 0.05).

**Figure 4:**
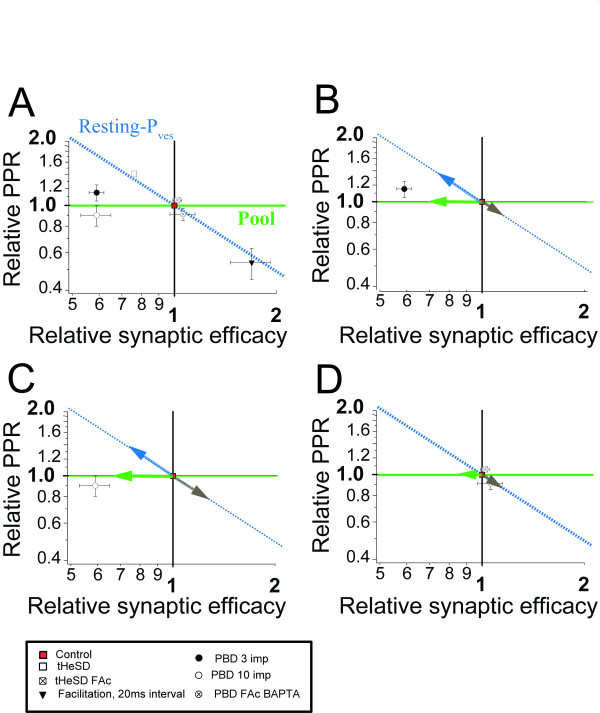
**Estimation of facilitation/augmentation and depletion following homosynaptic conditioning using the paired-pulse ratio**. *A*. Relationship between relative synaptic weight and relative PPR for different conditioning (indicated in the figure with different symbols) normalized with respect to control. Control synaptic weight and control PPR are set to 1. Dotted blue line indicates the expected relationship when there is a selective change of the first EPSP in the paired-pulse protocol (resting-P_ves_). Green horizontal line indicates the expected relationship when there are equal changes of the first and of the second EPSP (pool). Error bars for the tHeSD are within the symbol. *B*. Estimation of the contribution of "resting Pves" depression (blue arrow), facilitation/augmentation (grey arrow) and depletion (blue arrow) to the postburst depression 500 ms after a 3-impulse (50 Hz) burst. *C*. Same as *B*, but for the postburst depression 500 ms after a 10-impulse (50 Hz) burst. *D*. Same as *B*, but for the postburst depression 500 ms after a 3-impulse (50 Hz) burst when astrocyte signaling was compromised either by 50 mM BAPTA intracellularly, or 1 mM fluoroacetate extracellularly (Andersson and Hanse, 2010).

The results so far indicate that PBD is a variable mixture of three different forms of short-term plasticity; the astrocyte-mediated depression of "resting P_ves_", facilitation/augmentation and depletion of primed vesicles. Since these three forms short-term plasticity are associated with qualitatively very different changes in PPR, an analysis of the changes in PPR might be an alternative approach to estimate the reduction in the size of the primed vesicle pool by the homosynaptic conditioning. Figure 4A illustrates the relationship between changes in synaptic efficacy and changes in PPR for PBDs and tHeSDs. Since these changes are expressed as ratios they are plotted on logarithmic scales. Changes in Pr solely based on a change in the pool of primed vesicles are expected to affect EPSP1 and EPSP2 about equally and thus not result in changes of the PPR [13,19], as indicated by the green line. Changes in Pr based solely on a change in "resting Pves" are, on the other hand, expected to only affect EPSP1 thus resulting in reciprocal changes of the PPR, as indicated by the blue line. As discussed above, the tHeSD (open square) is a depression of "resting Pves" and its coordinates also fall close to the green line. When astrocyte metabolism was inhibited by FAc [12] the same conditioning stimuli results in no depression and no change in PPR (open square with cross). The coordinates for facilitation (filled triangle), and likely augmentation [18], are also close to the green line, indicating that astrocyte-mediated depression of "resting Pves" and facilitation/augmentation cause reciprocal changes of synaptic efficacy and PPR. The coordinates for PBD elicited by a 3-impulse burst (filled circle), by a 10-impulse burst (empty circle) and by a 3-impulse burst when astrocyte signaling was inhibited (open circle with cross, [11]) by either intracellular calcium chelation, or metabolic inhibition, are all relatively close to the green line.

To estimate how much a reduction in the pool contributes to the PBD elicited by a 3-impulse burst (Figure 4B), we assume that the three different short-term plasticities ("resting P_ves_" depression, facilitation/augmentation and depletion) multiply up to a net PBD. If "resting P_ves_" depression and facilitation/augmentation only occur in separate synapses (see also Discussion) one should instead have assumed an additive relationship. However, the quantitative difference between a multiplicative and additive relationship is not large using relevant magnitudes of these plasticities. For example, if "resting P_ves_" reduces synaptic efficacy to 0.75 and if facilitation/augmentation increases synaptic efficacy to 1.33 their combined action should be 1.0 using the multiplicative relationship and 1.08 using the additive relationship. The relationship between P_ves _and vesicle pool is likely sub-multiplicative [6,7], but for rather small absolute values of P_ves _and vesicle pool, and rather small changes in these parameters, a multiplicative relationship is a reasonable approximation. For example, if Pves is 0.2 and the number of primed, release ready, vesicles is 2, a reduction by a factor 2 of both these parameters will reduce P_r _from 0.36 to 0.1 (72%) using the sub-multiplicative relationship and from 0.4 to 0.1 (75%) using the multiplicative relationship, an error by about 4%.

Based on the increase of PPR in association with the tHeSD (1.41 ± 0.0024, n = 28) of control, Figure 1) and the full occlusion between the tHeSD and the PBD (Figure 2), we first estimate that the "resting P_ves_" depression contributes with 0.71 of control (1/1.41) to the PBD elicited by a 3-impulse burst (Figure 4B, blue arrow). Since PPR associated with the PBD was 1.15 ± 0.05, n = 28 of control and "resting P_ves_" depression should contribute by 1.41, facilitation/augmentation should contribute with a reduction in PPR by 0.82 (1.15/1.41), and an facilitation/augmentation associated with a PPR of 0.82 of control should correspond to an increase of the first EPSP (EPSC) by 1.22 (1/0.82, Figure 4B grey arrow). Having estimated the contribution from "resting P_ves_" depression (0.71) and from facilitation/augmentation (1.22) we estimated the contribution from depletion as the total PBD (0.59, ± 0.03, n = 28) divided by the product 0.71 and 1.22, yielding a value of 0.68 of control (32% depression, Figure 4B green arrow).

Using the same calculation we found that the estimated contribution of depletion to the PBD following the 10-impulse burst (Figure 4C) had increased to 0.54 (46% depression). With this method based on relative changes in PPR to estimate the depletion we thus find that depletion is 44% larger following the 10-impulse burst compared to that following the 3-impulse burst. This increased depletion is somewhat smaller, but comparable, to the increase we found using the method of cumulative burst response (51%, cf. Figure 3).

Compromising astrocyte signaling using either intracellular calcium chelation, or inhibition of the citric acid cycle resulted in a blockade of the PBD [11]. In fact, a small potentiation of 1.06 (± 0.09, n = 9) of control associated with a decrease of the PPR to 0.91 (± 0.06, n = 9) of control remained (Figure 4D). Since the "resting P_ves_" depression is expected to be to totally blocked under these conditions (Figure 4A) facilitation/augmentation alone should account for the change in PPR, that is, 1.10 (1/0.91). The estimated depletion under these conditions should then be 0.94 (6% depression), that is, substantially smaller than when astrocyte signaling was intact (32%, Figure 4B).

## Discussion

The present study has examined how astrocyte-mediated short-term depression affects release probability at glutamate synapses in the CA1 hippocampal region. Our main conclusions are that activation of astrocytes decreases vesicular release probability at inactive synapses ("resting P_ves_"), but not at active synapses ("active P_ves_"), and impose a delay in the recovery of primed vesicles following depletion by high-frequency activity.

### Depression of "resting P_ves_", but not "active P_ves_"

The tHeSD was associated with a selective depression of the first EPSP in a paired-pulse, or a high-frequency burst, protocol. The second and third EPSP evoked at 50 Hz were not depressed, but, if anything, slightly increased (Figure 1). This finding cannot be explained by a reduction in the number of release-ready (primed) vesicles, or by a general reduction in P_ves_, both of which are not expected to be restricted to only the first EPSP. It is also not consistent with reduced calcium influx which affects both EPSPs (although not uniformly) evoked by a paired-pulse stimulus [e.g. 20]. To explain the selective depression of the first EPSP we propose that "resting" and "active" P_ves _are differentially modulated such that astrocyte-mediated depression selectively affects "resting P_ves_". This proposal is in line with the finding that, although there is a large heterogeneity among developing hippocampal glutamate synapses regarding "resting P_ves_", "active P_ves_" is rather uniform among these synapses [9].

Although we are not aware of any previous description of P_r _modulation restricted to "resting P_ves_", this behavior is strikingly similar to the change in synaptic transmission produced by genetic elimination of the Rab3A-D [20]. Hippocampal synapses from these mice showed about 30% reduction in P_r _when tested with low frequency, but little, or no, reduction of P_r _when tested at high frequency. These findings from the Rab3-deficient mice indicated that Rab3s are involved in "superpriming" of vesicles in a subset of synapses [20]. Synapses with "superprimed" vesicles may well correspond to the subpopulation of high-P_r _synapses among the CA3-CA1 synapses [6,21-23]. Thus, a possible explanation for the tHeSD would be an astrocyte-mediated reversal of Rab3-dependent "superpriming" at high-Pr synapses. According to the time-course of tHeSD [12,24] this putative reversal of "superpriming" would develop during a few hundred ms and vanish within a few seconds.

### A combination of depression of "resting Pves", depletion of vesicles and augmentation during the PBD

The interaction between hetero- and homosynaptic conditioning indicated that the homosynaptic PBD consists of two separable components (Figure 2). One component is shared with the tHeSD and should then be expressed as a depression of "resting P_ves_" and an increased PPR, possibly related to reversal of Rab3-dependent "superpriming" at high-P_r _synapses. The other component is then specific for recently active synapses and seems to involve depletion of vesicles, contributing to further depression, and facilitation/augmentation, counteracting the depression.

That the conditioning burst causes depletion of vesicles available for release by the subsequent test stimulation was indicated by the analysis of the cumulative test train response (Figure 3). Another analysis, based on changes in PPR (Figure 4), supported this conclusion. This analysis was based on the premises that facilitation/augmentation is associated with decreased PPR [18] whereas depletion is associated with no, or very small, changes of PPR [13,19].

The presence of facilitation/augmentation was indicated by reduced PPR (and more so with longer conditioning trains) associated with the component of the PBD specific for the homosynaptic conditioning (Figure 4). In addition to the potentiation per se, facilitation/augmentation may contribute to counteract the depression (of the first EPSP) and to the decreased PPR in another way. Since facilitation/augmentation implies a shift from "resting P_ves_" to "active P_ves_", it will render synapses expressing it resistant to the astrocyte-mediated depression of "resting P_ves_". This scenario presupposes that facilitation/augmentation and the astrocyte-mediated depression of "resting P_ves_" can be expressed in the same synapse. As will be outlined below, this is, however, not likely the case. Augmentation is typically expressed at low-P_r _synapses and appears to rather specifically potentiate only the first EPSC in a high-frequency train, i.e. "resting P_ves_", [18] and is thus associated with a prominent decrease of the PPR [18,25]. The magnitude of augmentation increases with increased number of stimuli in the conditioning high-frequency stimulation, but its decay time constant is characteristically remarkably invariable, being 5-10 s [1]. Although it remains to be determined with more accuracy, the decay time constant of the astrocyte-mediated depression of "resting P_ves_" is in the order of about 1 s [12,24], possibly explaining the finding that facilitation/augmentation is larger 2 s, compared to 1 s, following a conditioning burst [17]. This finding is not consistent with astrocyte-mediated depression of "resting P_ves_" and augmentation occurring in the same synapses since, if they were, facilitation/augmentation would have precluded the expression of the "resting P_ves_" depression. In support for the idea that these two short-term synaptic plasticities occur in separate synapses our results indicated that, whereas facilitation/augmentation increased when increasing the number of stimuli in the conditioning train from 3 to 10, the depression of "resting P_ves_" did not increase, but remained at the same magnitude. As discussed above it is likely that augmentation occurs at low-P_r _synapses while astrocyte-mediated depression of "resting P_ves_" occurs at high-P_r _synapses.

We thus favor a scenario in which the conditioning burst elicits three different forms of short-term synaptic plasticity; a depression of "resting P_ves_" (possibly at high-P_r _synapses), facilitation/augmentation of "resting P_ves_" (possibly at low-P_r _synapses) and depletion of primed vesicles at all synapses. The relative contribution of these different forms of short-term plasticity will depend on the nature of the conditioning stimuli, on the conditioning - test interval, as well as on the relative number of low P_r _synapses in the synapse population.

### Astrocyte signaling imposes a delay in the recovery of primed vesicles

We have previously shown that inhibiting astrocyte metabolism and calcium signaling prevents the tHeSD and the PBD [11,12]. When analyzed in some more detail here (Figure 4), the prevention of the PBD was associated with decreased PPR, indicating the presence of facilitation/augmentation sufficient to just oppose depletion. Further analysis indicated that the depletion component was substantially reduced compared to the control situation, when astrocyte signaling was intact. The inhibition of astrocyte signaling did not affect the magnitude conditioning burst response, indicating that the size of pre-primed pool of vesicles as well as the depletion of vesicles per se by the conditioning burst is unaffected by astrocyte signaling. Therefore, to explain the larger pool 0.5 s after a conditioning burst we propose that recovery from depletion is faster when astrocyte signaling is inhibited, or conversely, that an astrocyte signal impose a delay of the re-priming following depletion. This proposal has a precedent from results at the Calyx of Held synapse at which activation of metabotropic glutamate autoreceptors slowed down the recovery after the burst, while not affecting the response during the continuous high-frequency activation [26]. To what extent activation of metabotropic glutamate receptors is involved in the astrocyte-mediated delay of re-priming after a conditioning burst will be examined in future studies.

Recovery of primed vesicles is thought to be mediated by a fast calcium-dependent (few hundred ms) and a slow calcium-independent (2-6 s) mechanism [8]. Increased presynaptic calcium concentration accelerates recovery of primed vesicles [27-31]. This mechanism relies on calmodulin [29] binding to Munc-13 [28], and is counteracted by presynaptic GABA_B _receptor activation reducing presynaptic cAMP levels [10]. Since the recruitment rate during a 10-impulse, 50 Hz, train following a 3-impulse burst was unaffected (Figure 3) it is unlikely that the presently proposed astrocyte-mediated delay of vesicle recovery acts by directly inhibiting the calcium-dependent priming. Therefore we propose that astrocyte signaling rather acts by slowing down calcium-independent recruitment, or by accelerating the decay of the calcium-dependent recruitment when stimulation stops (which is normally very rapid, in the order of 0.1 s [8]). As calcium-dependent recruitment can be modulated by the levels of cAMP [10,28] a putative glio-transmitter could mediate an acceleration of fast recruitment-decay by binding to a metabotropic receptor and decrease cAMP [32,33].

### Functional considerations

Our results suggest that a short burst activation of astrocytes results in a transient depression of "resting P_ves_" and a delay in the replenishment of primed vesicles after depletion. We propose that other mechanisms controlling P_r_, such as "active P_ves_", calcium-dependent recruitment and the size of release-ready primed vesicles, are not affected by the astrocyte signaling. These considerations may help to explain during which type of synaptic activity astrocyte-mediated short-term synaptic depression is operating. Thus, conditions during which there is residual calcium in the terminal, resulting in "active P_ves_" and calcium-dependent recruitment of new vesicles, are expected to preclude astrocyte-mediated depression. In line with this we found that continuous high-frequency trains were unaffected by inhibition of astrocyte signaling. We also found that continuous low-frequency trains were unaffected by inhibition of astrocyte signaling [11]. This finding is likely rather explained by insufficient temporal summation to activate the astrocytes. On the other hand, astrocyte-mediated short-term synaptic depression is expected to be prominently expressed during burst activity resembling theta burst activity (interburst frequencies of 2-10 Hz, intraburst frequencies > 50 Hz, and > 3 impulses in each burst) [34].

## Conclusion

Our results suggest that activated astrocytes depress the release probability via two different mechanisms; by depression of vesicular release probability only at inactive synapses and by imposing a delay in the recovery of the primed pool of vesicles following depletion. These mechanisms restrict the expression of the astrocyte-mediated depression to temporal windows that are typical for synaptic burst activity.

## Methods

### Slice preparation and solutions

Experiments were performed on hippocampal slices from 20- 50 day-old Wistar rats. The animals were killed in accordance with the guidelines of the local ethical committee for animal research (ref. 2008-2010-210). Rats were anaesthetized with isoflurane (Abbott) prior to decapitation. The brain was removed and placed in an ice-cold solution containing (in mM): 140 cholineCl, 2.5 KCl, 0.5 CaCl_2_, 7 MgCl_2_, 25 NaHCO_3_, 1.25 NaH_2_PO_4_, 1.3 ascorbic acid and 7 dextrose. Transverse hippocampal slices (300 - 400 μm thick) were cut with a vibratome (HM 650V Microm, Germany) in the same ice-cold solution. Slices were subsequently stored in artificial cerebrospinal fluid (ACSF) containing (in mM): 124 NaCl, 3 KCl, 2 CaCl_2_, 4 MgCl_2_, 26 NaHCO_3_, 1.25 NaH_2_PO_4_, 0.5 ascorbic acid, 3 myo-inositol, 4 D, L-lactic acid, and 10 D-glucose. After at least one hour of storage at 25° C, a single slice was transferred to a recording chamber where it was kept submerged in a constant flow (~2 ml min^-1^) at 30-32° C. The perfusion fluid contained (in mM) 124 NaCl, 3 KCl, 4 CaCl_2_, 4 MgCl_2_, 26 NaHCO_3_, 1.25 NaH_2_PO_4_, and 10 D-glucose. Picrotoxin (100 μM) was and D-AP5 (50 μM) was present in the perfusion fluid to block GABA_A _and NMDA receptor-mediated activity, respectively. All solutions were continuously bubbled with 95% O_2 _and 5% CO_2 _(pH ~7.4). The higher concentration of Ca^2+ ^and Mg^2+ ^than normal was used to inhibit network activity. In a subset of experiments a cut was made between area CA3 and area CA1. However, since we observed the same amount depression in slices with and without a cut, the data from these two sets of experiments were pooled.

### Field recordings and analysis

Electrical stimulation of Schaffer collateral/commissural axons and recordings of synaptic responses were carried out in the stratum radiatum of the CA1 region. Stimuli consisted of biphasic constant current pulses (15 - 80 μA, 200 μS, STG 1002 Multi-Channel Systems MCS Gmbh, Reutlingen, Germany) delivered through tungsten wires (resistance ~0.1 MΩ,). One stimulation electrode was positioned in the stratum radiatum with a distance of 100 μm from the registration electrode (Figure 1A). The synaptic input was activated every 10 s and stimulation intensity was adjusted so that spike activity was observed on the second or third fEPSP (but not on the first fEPSP) in the conditioning train. Field EPSPs were recorded with a glass micropipette (filled with perfusion fluid or 1 M NaCl, resistance 1-2 MΩ). Field EPSPs were sampled at 10 kHz with an EPC-9 amplifier (HEKA Elektronik, Lambrecht, Germany) and filtered at 1 kHz. Evoked responses were analyzed off-line using custom-made IGOR Pro (WaveMetrics, Lake Oswego, OR) software. Field EPSP magnitude was estimated by linear regression over the first 0.8 ms of the initial slope. Paired-pulse ratio (PPR) was calculated as the ratio of the initial slope of the EPSP2 (EPSC2) divided by the initial slope of the EPSP1 (EPSC1). The initial slope was measured using linear regression of only the first 0.8 ms of the EPSP (EPSC) initial slope to avoid possible influence by spike activity. GABA_A _receptors were blocked by picrotoxin precluding influence of GABA_A _receptor mediated IPSPs (IPSCs) on the PPR. Although a change in PPR is widely used as an indicator of a change in Pr [1,19], PPR can change without a change in Pr. For example, changes in synaptic efficacy (including silencing/unsilencing) caused by mechanisms other than a change in Pr, but only in a subpopulation of synapses whose mean Pr differs from the mean Pr of the synapse population recorded from, will change the PPR [cf. 35]. Moreover, postsynaptic mechanisms including AMPA receptor saturation, desensitization or polyamine unblock of calcium permeable AMPA receptors may contribute to changes in PPR, but such contributions are thought be negligible at CA3-CA1 synapses using interstimulus intervals of 20 ms, or longer [36-41]. The presynaptic volley was measured as the slope of the initial positive-negative deflection, and it was not allowed to change by more than 10% during the experiment.

### Patch clamp recordings pyramidal cells and astrocytes

Pyramidal cells were visually identified in the CA1 area with IR-DIC video microscopy (Nikon) and patched with an intracellular solution containing (in mM): 120 Cs-methane sulphonate, 2 NaCl, 10 HEPES, 5 Qx-314, 4 Mg-ATP, 0.4 GTP and 20 BAPTA (pH ~7, 2 and osmolality to 295-300 mosmol). For measuring AMPA responses the cell was held at - 80 mV. Evoked responses were analyzed off-line using IGOR Pro (WaveMetrics, Lake Oswego, OR) software. Amplitudes were measured on average-sweeps of 18 consecutive sweeps. Astrocytes in the stratum radiatum were identified by their small soma and, when patched, for their linear responses to voltage steps [42]. The intracellular solution contained (in mM): 120 KCl, 2 NaCl, 20 HEPES, 4 Mg-ATP, 0.4 GTP and 50 BAPTA. Patch pipettes (1·5 mm/0·86 mm; borosilicate, Clark Electrochemical Instruments) were pulled with a horizontal puller (Sutter Instruments Inc.) to a resistance of 3-6 MΩ. Series resistance was measured using a 5 ms, 10 mV hyperpolarising pulse and was not allowed to change more than 15% during the experiment.

Data are expressed as means ± SEM. Statistical significance for paired and independent samples was evaluated using Student's t - test.

### Drugs

Drugs were from Tocris Cookson (Bristol, UK) except picrotoxin and fluoroacetate from Sigma-Aldrich (Stockholm, Sweden) and D-AP5 from Accent Scientific.

## Authors' contributions

All authors read and approved the final manuscript. MA participated in experiment design, performed the experiments, analyzed the data and prepared the manuscript. EH conceived of the study, and participated in its design and preparation of the manuscript.

## References

[B1] Zucker RS Regehr WG Short-term synaptic plasticity Annu Rev Physiol 2002 64 355 405 10.1146/annurev.physiol.64.092501.114547 11826273

[B2] Pinheiro PS Mulle C Presynaptic glutamate receptors: physiological functions and mechanisms of action Nat Rev Neurosci 2008 9 6 423 436 18464791 10.1038/nrn2379

[B3] MacDermott AB Role LW Siegelbaum SA Presynaptic ionotropic receptors and the control of transmitter release Annu Rev Neurosci 1999 22 443 485 10.1146/annurev.neuro.22.1.443 10202545

[B4] Miller RJ Presynaptic receptors Annual review of pharmacology and toxicology 1998 38 201 227 10.1146/annurev.pharmtox.38.1.201 9597154

[B5] Branco T Staras K The probability of neurotransmitter release: variability and feedback control at single synapses Nat Rev Neurosci 2009 10 5 373 383 10.1038/nrn2634 19377502

[B6] Hanse E Gustafsson B Vesicle release probability and pre-primed pool at glutamatergic synapses in area CA1 of the rat neonatal hippocampus J Physiol 2001 531 Pt 2 481 493 11230520 10.1111/j.1469-7793.2001.0481i.x PMC2278469

[B7] Sakaba T Schneggenburger R Neher E Estimation of quantal parameters at the calyx of Held synapse Neurosci Res 2002 44 4 343 356 10.1016/S0168-0102(02)00174-8 12445623

[B8] Neher E Sakaba T Multiple roles of calcium ions in the regulation of neurotransmitter release Neuron 2008 59 6 861 872 10.1016/j.neuron.2008.08.019 18817727

[B9] Hanse E Gustafsson B Factors explaining heterogeneity in short-term synaptic dynamics of hippocampal glutamatergic synapses in the neonatal rat J Physiol 2001 537 Pt 1 141 149 11711568 10.1111/j.1469-7793.2001.0141k.x PMC2278933

[B10] Sakaba T Neher E Direct modulation of synaptic vesicle priming by GABA(B) receptor activation at a glutamatergic synapse Nature 2003 424 6950 775 778 10.1038/nature01859 12917685

[B11] Andersson M Hanse E Astrocytes impose postburst depression of release probability at hippocampal glutamate synapses J Neurosci 2010 30 16 5776 5780 10.1523/JNEUROSCI.3957-09.2010 20410129 PMC6632337

[B12] Andersson M Blomstrand F Hanse E Astrocytes play a critical role in transient heterosynaptic depression in the rat hippocampal CA1 region J Physiol 2007 585 Pt 3 843 852 17962333 10.1113/jphysiol.2007.142737 PMC2375509

[B13] Abrahamsson T Gustafsson B Hanse E Synaptic fatigue at the naive perforant path-dentate granule cell synapse in the rat J Physiol 2005 569 Pt 3 737 750 16239273 10.1113/jphysiol.2005.097725 PMC1464272

[B14] Schneggenburger R Meyer AC Neher E Released fraction and total size of a pool of immediately available transmitter quanta at a calyx synapse Neuron 1999 23 2 399 409 10.1016/S0896-6273(00)80789-8 10399944

[B15] Stevens CF Wesseling JF Augmentation is a potentiation of the exocytotic process Neuron 1999 22 1 139 146 10.1016/S0896-6273(00)80685-6 10027296

[B16] Garcia-Perez E Wesseling JF Augmentation controls the fast rebound from depression at excitatory hippocampal synapses J Neurophysiol 2008 99 4 1770 1786 10.1152/jn.01348.2007 18199812

[B17] Gustafsson B Asztely F Hanse E Wigstrom H Onset Characteristics of Long-Term Potentiation in the Guinea-Pig Hippocampal CA1 Region in Vitro Eur J Neurosci 1989 1 4 382 394 10.1111/j.1460-9568.1989.tb00803.x 12106147

[B18] Granseth B Lindstrom S Augmentation of corticogeniculate EPSCs in principal cells of the dorsal lateral geniculate nucleus of the rat investigated in vitro J Physiol 2004 556 Pt 1 147 157 14724203 10.1113/jphysiol.2003.053306 PMC1664880

[B19] Hanse E Gustafsson B Paired-pulse plasticity at the single release site level: an experimental and computational study J Neurosci 2001 21 21 8362 8369 11606624 10.1523/JNEUROSCI.21-21-08362.2001 PMC6762810

[B20] Schluter OM Basu J Sudhof TC Rosenmund C Rab3 superprimes synaptic vesicles for release: implications for short-term synaptic plasticity J Neurosci 2006 26 4 1239 1246 10.1523/JNEUROSCI.3553-05.2006 16436611 PMC6674574

[B21] Dobrunz LE Stevens CF Heterogeneity of release probability, facilitation, and depletion at central synapses Neuron 1997 18 6 995 1008 10.1016/S0896-6273(00)80338-4 9208866

[B22] Hessler NA Shirke AM Malinow R The probability of transmitter release at a mammalian central synapse Nature 1993 366 6455 569 572 10.1038/366569a0 7902955

[B23] Rosenmund C Clements JD Westbrook GL Nonuniform probability of glutamate release at a hippocampal synapse Science 1993 262 5134 754 757 10.1126/science.7901909 7901909

[B24] Isaacson JS Solis JM Nicoll RA Local and diffuse synaptic actions of GABA in the hippocampus Neuron 1993 10 2 165 175 10.1016/0896-6273(93)90308-E 7679913

[B25] McNaughton BL Long-term synaptic enhancement and short-term potentiation in rat fascia dentata act through different mechanisms J Physiol 1982 324 249 262 7097600 10.1113/jphysiol.1982.sp014110 PMC1250703

[B26] Billups B Graham BP Wong AY Forsythe ID Unmasking group III metabotropic glutamate autoreceptor function at excitatory synapses in the rat CNS J Physiol 2005 565 Pt 3 885 896 15845577 10.1113/jphysiol.2005.086736 PMC1464548

[B27] Dittman JS Regehr WG Calcium dependence and recovery kinetics of presynaptic depression at the climbing fiber to Purkinje cell synapse J Neurosci 1998 18 16 6147 6162 9698309 10.1523/JNEUROSCI.18-16-06147.1998 PMC6793194

[B28] Junge HJ Rhee JS Jahn O Varoqueaux F Spiess J Waxham MN Rosenmund C Brose N Calmodulin and Munc13 form a Ca2+ sensor/effector complex that controls short-term synaptic plasticity Cell 2004 118 3 389 401 10.1016/j.cell.2004.06.029 15294163

[B29] Sakaba T Neher E Calmodulin mediates rapid recruitment of fast-releasing synaptic vesicles at a calyx-type synapse Neuron 2001 32 6 1119 1131 10.1016/S0896-6273(01)00543-8 11754842

[B30] Stevens CF Wesseling JF Activity-dependent modulation of the rate at which synaptic vesicles become available to undergo exocytosis Neuron 1998 21 2 415 424 10.1016/S0896-6273(00)80550-4 9728922

[B31] Wang LY Kaczmarek LK High-frequency firing helps replenish the readily releasable pool of synaptic vesicles Nature 1998 394 6691 384 388 10.1038/28645 9690475

[B32] Allen NJ Barres BA Signaling between glia and neurons: focus on synaptic plasticity Curr Opin Neurobiol 2005 15 5 542 548 10.1016/j.conb.2005.08.006 16144764

[B33] Santello M Volterra A Synaptic modulation by astrocytes via Ca2+-dependent glutamate release Neuroscience 2009 158 1 253 259 10.1016/j.neuroscience.2008.03.039 18455880

[B34] Lisman J Buzsaki G A neural coding scheme formed by the combined function of gamma and theta oscillations Schizophr Bull 2008 34 5 974 980 10.1093/schbul/sbn060 18559405 PMC2518638

[B35] Poncer JC Malinow R Postsynaptic conversion of silent synapses during LTP affects synaptic gain and transmission dynamics Nat Neurosci 2001 4 10 989 996 10.1038/nn719 11544481

[B36] Hanse E Gustafsson B Quantal variability at glutamatergic synapses in area CA1 of the rat neonatal hippocampus J Physiol 2001 531 Pt 2 467 480 11230519 10.1111/j.1469-7793.2001.0467i.x PMC2278484

[B37] Hjelmstad GO Isaac JT Nicoll RA Malenka RC Lack of AMPA receptor desensitization during basal synaptic transmission in the hippocampal slice J Neurophysiol 1999 81 6 3096 3099 10368425 10.1152/jn.1999.81.6.3096

[B38] Liu G Choi S Tsien RW Variability of neurotransmitter concentration and nonsaturation of postsynaptic AMPA receptors at synapses in hippocampal cultures and slices Neuron 1999 22 2 395 409 10.1016/S0896-6273(00)81099-5 10069344

[B39] McAllister AK Stevens CF Nonsaturation of AMPA and NMDA receptors at hippocampal synapses Proc Natl Acad Sci USA 2000 97 11 6173 6178 10.1073/pnas.100126497 10811899 PMC18577

[B40] Adesnik H Nicoll RA Conservation of glutamate receptor 2-containing AMPA receptors during long-term potentiation J Neurosci 2007 27 17 4598 4602 10.1523/JNEUROSCI.0325-07.2007 17460072 PMC6672988

[B41] Stubblefield EA Benke TA Distinct AMPA-type glutamatergic synapses in developing rat CA1 hippocampus J Neurophysiol 2010 104 4 1899 1912 10.1152/jn.00099.2010 20685930 PMC2957466

[B42] Matthias K Kirchhoff F Seifert G Huttmann K Matyash M Kettenmann H Steinhauser C Segregated expression of AMPA-type glutamate receptors and glutamate transporters defines distinct astrocyte populations in the mouse hippocampus J Neurosci 2003 23 5 1750 1758 12629179 10.1523/JNEUROSCI.23-05-01750.2003 PMC6741945

